# Long-Term Survival Rate of Autogenous Tooth Transplantation: Up to 162 Months

**DOI:** 10.3390/medicina58111517

**Published:** 2022-10-25

**Authors:** Jin-Han Park, Yong-Gun Kim, Jo-Young Suh, Myoung-Uk Jin, Jae-Mok Lee

**Affiliations:** 1 Department of Periodontology, Kyungpook National University School of Dentistry, Daegu 41940, Korea; 2 Department of Conservative Dentistry, Kyungpook National University School of Dentistry, Daegu 41940, Korea

**Keywords:** autogenous tooth transplantation, long-term survival rate, pre-implant treatment

## Abstract

*Background and Objectives*: The purpose of this study is to observe the usefulness of autogenous tooth transplantation by examining the cumulative survival rate according to the period of auto-transplanted teeth as pre-implant treatment. *Materials and Methods*: This study was conducted on 111 patients who visited Kyungpook National University Dental Hospital and underwent autogenous tooth transplantation between November 2008 and January 2021 (about 13 years). The cumulative survival rate of autogenous tooth transplantation according to the causes of extraction of the recipient tooth (caries, periapical lesion, crack, crown fracture, periodontitis) and condition of opposing teeth (natural teeth vs. fixed prosthesis). The cumulative survival rate of autogenous tooth transplantation according to the age (under 30 vs. over 30) was also investigated and it was examined whether there were any differences in each factor. *Results*: The average follow-up period was 12 months, followed by a maximum of 162 months. The 24-month cumulative survival rate of all auto-transplanted teeth was 91.7%, 83.1% at 60 months and the 162-month cumulative survival rate was 30.1%. There were no statistical differences between the causes of extraction of the recipient’s teeth, differences in the condition of the opposing teeth, and differences under and over the age of 30. *Conclusions*: The survival rate of autogenous tooth transplantation appears to be influenced by the conditions of the donor tooth rather than the conditions of the recipient tooth. Although autogenous tooth transplantation cannot completely replace implant treatment, it is meaningful in that it can slightly delay or at least earn the time until implant placement is possible.

## 1. Introduction

When a tooth is lost, several treatments can be considered to reconstruct the lost tooth. In modern society, the most common way to reconstruct lost teeth is implant procedures, but in addition to implants, bridge and denture treatment, autogenous tooth transplantation can be considered, which is transplanting one tooth within the same patient to another location [1]. Autogenous tooth transplantation, introduced by Hale in 1954, can be used when other treatments cannot be performed due to the patient’s financial reasons or age [2,3]. In the case of implants, they cannot be used at young ages who have not yet completed growth because implants do not follow the patient’s craniofacial development, but auto-transplanted teeth have the advantage of being able to develop with the patient’s growth [4]. Auto-transplanted teeth are less likely to cause immune rejection response, and it has the advantage of being connected and communicating with the human body, and it can be expected to maintain the volume of alveolar bone or to regenerate the alveolar bone through physiological stimulation of the periodontal ligament (PDL) [5,6,7,8]. Regeneration of alveolar bone can also have a positive effect on implant placement after losing auto-transplanted teeth [9,10]. Also, since there is a possibility that some dental implants with recurrent peri-implantntis should be removed within a few years, autogenous tooth transplantation has the advantage of delaying the implant placement and provide physiological tooth replacement [4]. Compared to conventional implants, it has the ability to defend itself against inflammatory reactions [7,11]. Indications for autogenous tooth transplantation include loss due to periodontitis, failure of root canal treatment, impacted or ectopic tooth, tooth fracture or loss due to premature or traumatic causes, tooth loss due to tumor or iatrogenic causes, inter-arch length discrepancy, missing tooth, and replacement of poor prognosis tooth [12,13,14]. In addition, it provides a proprioceptive sensation [15].

However, there are also many things to be considered. In order for the donor site to be extracted without damage, the degree of impaction of the donor site, the shape of the root, and the integrity of the donor tooth must be considered [16,17,18,19]. For stable transplantation at the recipient site, the proximal-distal size of the recipient and the shape of the alveolar bone must be in harmony with the donor teeth, and the alveolar bone of the recipient should be sufficiently present or at least enough for stable support of the donor tooth when it splinted with adjacent teeth [20,21]. Also, root canal treatment and crown restoration should be additionally performed. Furthermore, the time and economic aspects required for this are also factors to be considered. The purpose of this study is to observe the usefulness of autogenous tooth transplantation by examining the survival rate according to the survival period of auto-transplanted teeth. The null hypothesis of the present study was that there is no significant difference in the survival rate according to the condition of the recipient sites.

## 2. Materials and Methods

The research protocol of this study was reviewed and approved by the Research Ethics Committee, Kyungpook National University (KNUDH 2021-06-03-00).

This study was conducted on 111 patients who visited Kyungpook National University Dental Hospital and underwent autogenous tooth transplantation between November 2008 and January 2021 (about 13 years). Patients who underwent one or more autogenous tooth transplantations were included. When existing teeth were lost due to pathological causes such as caries, fractures, periodontitis, and apical lesions, the recipient site was reconstructed using the 3rd molar mainly as a donor site.

Prior to the operation, an individual’s underlying disease was investigated, and patients with systemic diseases that increase the risk of surgery were excluded from the operation. Clinical and radiological examination were performed before and after autogenous tooth transplantation. Additional clinical and radiological examination were performed if necessary. After clinical and radiological examination, periodontal treatment, endodontic treatment and crown restoration were performed if necessary. 

**Figure 1:**
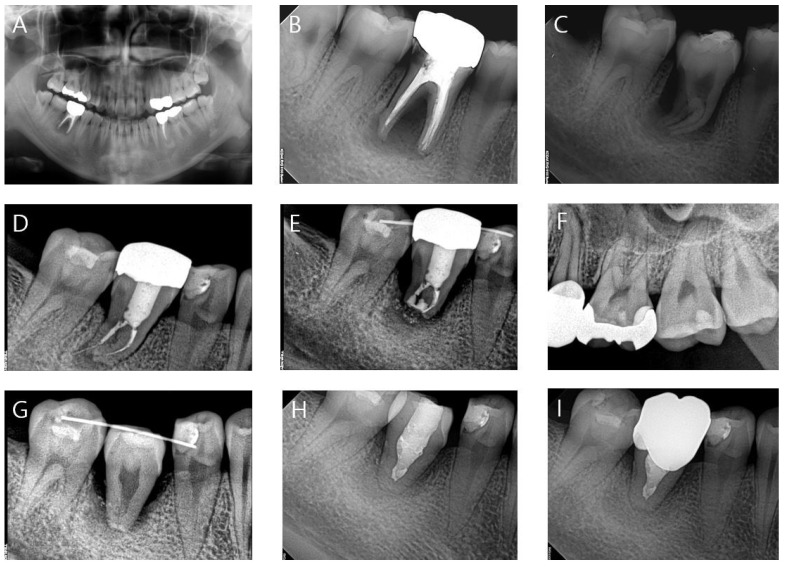
(**A**,**B**) Panoramic and periapical view at first visit. Periapical view immediately (**C**) and 7 years (**D**) after first autogenous tooth transplantation from #48 to #46. (**E**) 2 months after root resection (**F**). Periapical view of donor site (#28). Periapical view after 1 week (**G**), 3 months (**H**) and 18 months (**I**) after second autogenous tooth transplantation from #28 to #46.

All patients were operated according to the same protocol, and prophylactic antibiotics were not prescribed. Figure 1 shows the overall procedure of treatment using autogenous tooth transplantation.

A 22-year-old patient (Figure 1) presented with primary endodontic with secondary periodontal involvement (A,B). Clinician decided to perform autogenous tooth transplantation from #48 to #46 with agreement of patient (C). Endodontic treatment and crown restoration were performed after 2 weeks. After that, the patient could not visit the clinic for 7 years due to personal reasons. 7 years later, she visited the clinic for periapical lesion on #46 (D). Root resection treatment was performed but periapical lesion spread to the periodontal tissue, and tooth could not be saved (E). As 7 years ago, clinician decided to perform autogenous tooth transplantation from #28 (F) to #46. Since the initial stability was not adequate, teeth splinting was performed using resin and wire (G). It remained stable until 3 months later, followed by endodontic treatment (H). The clinical result showed stable until 18 months after surgery, and regeneration of alveolar bone was confirmed (I).

### 2.1. Surgical Technique

The procedure was performed under local anesthesia, and the recipient site tooth was first extracted. After that, wet gauze was applied to prevent the recipient site from drying out. The teeth at the donor site were carefully removed using forceps, and saline irrigation was continued throughout the operation to prevent the cementum and PDL from drying out. The alveolar bone of the recipient site was appropriately prepared with a highspeed handpiece and round bur to allow the donor tooth to enter with an appropriate fit. If necessary, rotate it slightly to insert the donor tooth into the recipient site. After that, the suture was performed, and if the initial fixation was not made enough, it was attached using a resin splint with adjacent teeth. 

### 2.2. Statistical Methods

The cumulative survival rate of auto-transplanted teeth was investigated, and the Kaplan Meier survival curve was measured using the SPSS statistical program. In addition, the cumulative survival rate according to the cause of tooth extraction, the patient’s age (under 30 vs. over 30 years old), the condition of the opposing tooth (natural teeth vs. fixed prosthesis) was examined and Kaplan Meier survival curve was used to confirm significant differences between each group. 

## 3. Results

A total of 111 autogenous tooth transplantation were performed in 111 patients. There were 56 males and 55 females, with an average age of 36.5 years, with a minimum age of 13 and a maximum of 65 years (Table 1). 

The distribution of recipient and donor teeth is shown in Table 2. 94% of donor teeth were third molars, and they were transplanted as first and second molars. One incisor and two premolars were transplanted to incisor and premolars, respectively.

The average follow-up period was about 12 months and follow-up ranged from a minimum of 1 month to a maximum of 162 months. Of the 111 donor teeth, 104 were third molar tooth, and 108 of the 111 recipient teeth were molar sites. A total of 14 teeth out of 111 teeth were extracted, and the remaining 97 teeth are either survive or remain in a state that cannot be investigated further due to follow up loss. 

**Figure 2:**
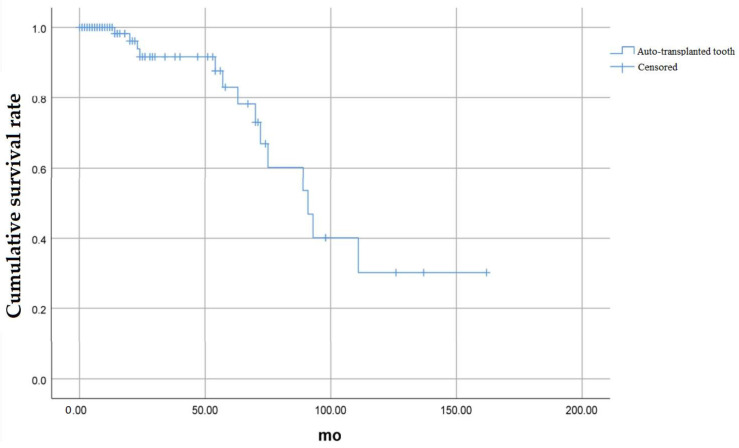
Overall cumulative survival rate of auto-transplanted tooth.

Figure 2 shows the overall cumulative survival rate for the auto-transplanted tooth, and the cumulative survival rate at 24 months was 91.7%. The final survival rate at 162 months was 30.1%.

### 3.1. Cumulative Survival Rate According to the Cause of Extraction of the Recipient Teeth

**Figure 3:**
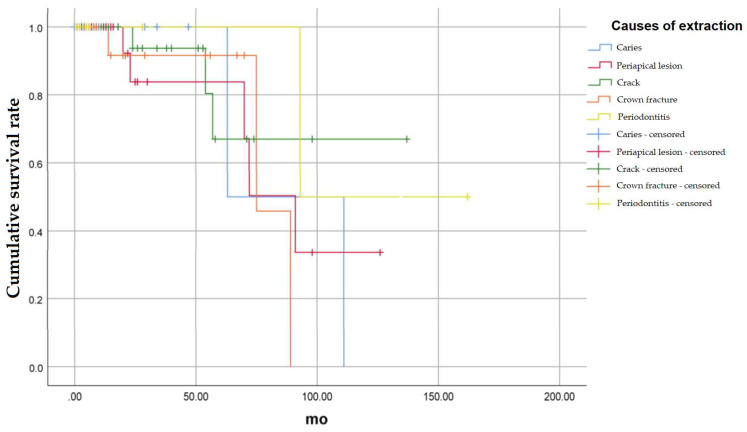
Cumulative survival rate of auto-transplanted tooth according to the cause of extraction of the recipient teeth.

The survival rate according to the cause of extraction of the recipient teeth was compared (Figure 3). The causes of tooth extraction were divided into 5 categories: Caries, Periapical lesion, Crack, Crown Fracture, and Periodontitis.

#### 3.1.1. Caries

There was a total of 21 cases in which the cause of tooth extraction was caries, of which two teeth were extracted. The 24-month cumulative survival rate was 100%, 50% at 63 months, and 0% at 111 months (*p* = 0.772).

#### 3.1.2. Periapical Lesion

There were a total of 35 cases in which the cause of extraction was periapical lesion, of which 5 teeth were extracted. The 24-month cumulative survival rate was 83.9%, 50.3% at 72 months, and 33.6% at 126 months (*p* = 0.576).

#### 3.1.3. Crack

There were a total of 30 cases in which the cause of tooth extraction was crack, and three of them were extracted. The 24-month cumulative survival rate was 93.8%, 80.4% at 54 months, and 67.0% at 57 and 137 months (*p* = 0.555).

#### 3.1.4. Crown Fracture

There were a total of 17 cases in which the cause of tooth extraction was crown fracture, of which three teeth were extracted. The cumulative survival rate at 24 months was 91.7%, 45.8% at 75 months, and 0% at 89 months (*p* = 0.560).

#### 3.1.5. Periodontitis

There were a total of 8 cases in which the cause of tooth extraction was periodontitis, of which one tooth was extracted. The 24-month cumulative survival rate was 100%, 50% at 93 months, and 50.0% at 162 months (*p* = 0.407).

There was no statistically significant difference in the survival rate according to the cause of tooth extraction.

### 3.2. Cumulative Survival Rate According to the Condition of Opposing Teeth

**Figure 4:**
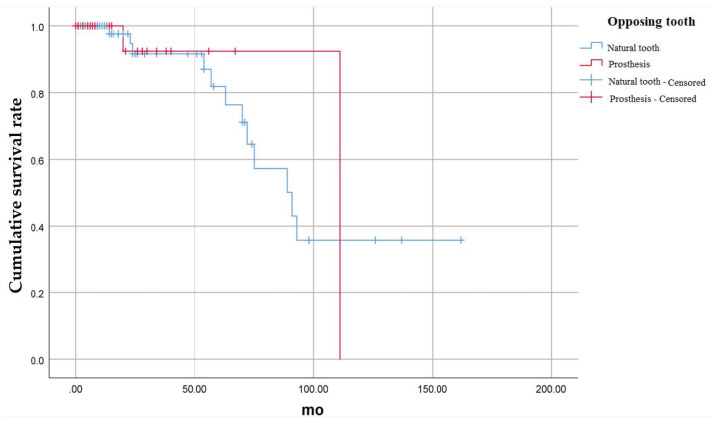
Cumulative survival rate of auto-transplanted tooth according to the condition of opposing teeth.

The difference in the cumulative survival rate according to whether the opposing tooth was a natural tooth, or a fixed prosthesis was also investigated (Figure 4).

There were a total of 87 cases in which the opposing tooth was a natural tooth, and a total of 24 cases in which the opposing tooth was treated with fixed prosthesis. If the opposing tooth was a natural tooth, a total of 12 teeth were extracted during the investigation period. If the opposing tooth was a fixed prosthesis, a total of 2 teeth were extracted during the investigation period.

When the opposing tooth was a natural tooth, the 24-month cumulative survival rate was 91.5%, 81.8% at 57 months, 70.9% at 70 months, and 35.8% at 162 months. In case of fixed prosthesis, the cumulative survival rate of 24 months and 67 months was 92.3%, but the cumulative survival rate of 111 months was 0%. There was no statistically significant difference in the cumulative survival rate between the two groups (*p* = 0.831).

### 3.3. Cumulative Survival Rate According to the Age of Patients

**Figure 5:**
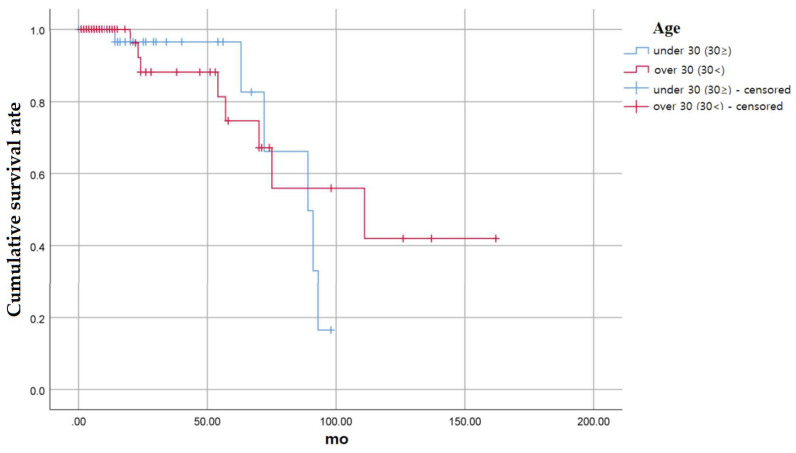
Cumulative survival rate of auto-transplanted tooth according to the age of patients.

We also compared the survival rate according to the age at the time of tooth extraction (Figure 5). 

The cumulative survival rate was measured by dividing into two groups: a group under 30 years old and a group over 30 years old. There were a total of 47 cases in the group under 30 years old, of which six teeth were extracted. There were a total of 64 cases in the over 30 group, of which 8 teeth were extracted.

In the group under 30 years of age, the 24-month cumulative survival rate was 96.4%, 82.7% at 63 months, 66.1% at 72 months, 49.6% at 89 months, 33.1% at 91 months, and 16.5% at 98 months.

In the group over 30 years of age, the 24-month cumulative survival rate was 88.1%, 81.4% at 54 months, 74.6% at 57 months, 67.1% at 70 months, 55.9% at 75 months, 42.0% at 111 months, and 42.0% at 162 months. There was no statistically significant difference between the two groups (*p* = 0.692).

Table 3 shows the cumulative survival rate and significance level according to various factors and periods. If there was no additional follow up, it is shown as a blank space.

## 4. Discussion

The purpose of this study was to investigate the survival rate according to the period after autogenous tooth transplantation. The null hypothesis was that there is no difference in the survival rate according to various conditions of recipient sites. Based on the study results, the null hypothesis was accepted (*p* > 0.05; Table 3). This can be considered as a problem of alveolar bone regeneration and the integration between alveolar bone and teeth, which are caused by periodontal ligament. Attachment of viable PDL to the donor site after treatment is important for the success of autogenous tooth transplantation. PDL-resident stem cells can differentiate into three types of cells: fibroblast, cementoblast and osteoblast. Differentiated osteoblast can induce surrounding alveolar bone which can provide adequate support, leading to stable maintenance and function of tooth. With the long-term survival rate of auto-transplanted teeth, PDL plays an important role in bone remodeling [22,23,24]. In the aspect of periodontal ligament, it is a factor determined by donor site, and it can be inferred that there was no statistically significant difference because the experimental factors of this study differ according to the status of the recipient site. In addition, it can be inferred that the cause of extraction of the recipient site does not significantly affect the integration of the donor tooth.

In addition to this, factors that can affect the survival rate can be considered the degree of openness of the donor tooth apex [25,26,27]. Invasion of micro vessels into the pulp through the apical foramen causes revascularization and provides nutrients with the blood supply [28]. The pulp healing occurs more easily as the openness of the apical foramen gets increased. When the diameter of the apical foramen is more than 1 mm, about 89% of the pulpal healing can be expected [29]. Therefore, the ideal indication for autogenous tooth transplantation is stage 3–4 of the Moorrees classification, which is about 50–75% root formation of the expected complete root development. However, in this study, the average age of the patients was 36.5 years old with stage 7 of the Moorrees classification; most of them have closed apical foramen. It caused limited pulp healing and, eventually, a low cumulative survival rate of teeth. 

Loss of more than half of patients during the follow-up was significant limitations within the present study. Of the total 111 patients, 62 patients were lost to follow up, representing a decrease in the number of subjects that can be investigated. As a result, there is often a rapid decrease in the survival rate after 5 years in this study; it acts as a bias in the cumulative survival rate. If more transplanted teeth that were included were continuous during the observation period, the higher cumulative survival rate of transplanted tooth would be expected. Therefore, further study should include more extensive subjects and a longer period of time for data collection.

The survival rate of autogenous tooth transplantation is lower than that of dental implants [30]. In spite of that, transplanted autogenous tooth has advantages over implants in terms of function, esthetic, cost, and time [22,27,31,32]. It establishes a physiological connection by PDL stimulation. Since it enables bone regeneration even with bone defect and presents faster healing, bone augmentation therapy with the usage of barrier membrane and bone graft would be no longer necessary. In addition, as the eruption process is not undergone with osseointegrated implant, infraocclusion and esthetic/functional problems may occur, especially in younger patients. However, auto-transplanted teeth can be erupted harmoniously with adjacent and opposing teeth, as well as it is possible to perform postoperative orthodontic treatment [22,27,31,32,33,34]. On top of that, compared to implant, esthetic harmony can be achievable with proper crown shape, color of natural teeth and a proper emergence profile. Therefore, with the presence of an appropriate donor teeth, autogenous tooth transplantation should be considered prior to other treatments.

## 5. Conclusions

Since the 5-year survival rate is 83.1%, autogenous tooth transplantation may be considered in case of tooth loss to delay implant or bridge treatment. By performing autogenous tooth transplantation, oral reconstruction using one’s own teeth can be attempted, and periodontal tissue regeneration can be induced, which can have a positive effect on future implant placement. In addition, it can be confirmed that similar results are obtained even when autogenous tooth transplantation is performed regardless of various conditions, such as the causes of extraction of the recipient, the condition of the opposing tooth or age of patient. Therefore, regardless of the status of the recipient site, clinicians may consider autogenous tooth transplantation when appropriate donor teeth are available.

## Figures and Tables

**Table 1:** Characteristics of patients.

Sex	Number	Age (Years)	Age Range
All	111	36.5 ± 13.0	13–65
Male	56 (50.5)	39.3 ± 13.9	13–63
Female	55 (49.5)	33.7 ± 11.7	15–65

Values are presented as number (%) or mean ± standard deviation.

**Table 2:** Distribution of donor and recipient teeth.

	Recipient Site	Donor Site
Incisor	1 (1)	1 (1)
Canine	0	0
Premoalr	2 (2)	2 (2)
First Molar	48 (43)	0
Second Molar	60 (54)	4 (3)
Third Molar	0	104 (94)

Values are presented as number (%).

**Table 3:** Cumulative survival rate (%) based on multiple variables.

Variable	Overall	Causes of Extraction					Opposing Teeth		Age	
	Overall (*n* = 111)	Caries (*n* = 21)	Periapical Lesion (*n* = 35)	Crack (*n* = 30)	Crown Fracture (*n* = 30)	Periodontitis (*n* = 8)	Natural Teeth (*n* = 87)	Prosthesis (*n* = 24)	Under 30 (*n* = 47)	Over 30 (*n* = 64)
Period (year)										
1	100.0	100.0	100.0	100.0	100.0	100.0	100.0	100.0	100.0	100.0
2	91.7	100.0	83.9	93.8	91.7	100.0	91.5	92.3	96.4	88.1
3	91.7	100.0	83.9	93.8	91.7	100.0	91.5	92.3	96.4	88.1
4	91.7	100.0	83.9	93.8	91.7	100.0	91.5	92.3	96.4	88.1
5	83.1	100.0	83.9	67.0	91.7	100.0	81.8	92.3	96.4	74.6
6	66.9	50.0	50.3	67.0	91.7	100.0	64.5	92.3	66.1	67.1
7	60.2	50.0	50.3	67.0	45.8	100.0	57.3	92.3	66.1	55.9
8	40.1	50.0	33.6	67.0	0.0	50.0	35.8	92.3	16.5	55.9
9	40.1	50.0	33.6	67.0		50.0	35.8	92.3		55.9
10	30.1	0.0	33.6	67.0		50.0	35.8	0.0		42.0
11	30.1		33.6	67.0		50.0	35.8			42.0
12	30.1			67.0		50.0	35.8			42.0
13	30.1					50.0	35.8			42.0
*p*-value		0.772	0.576	0.555	0.560	0.407	0.831		0.692	

## Data Availability

The datasets used and/or analyzed during the current study are available from the corresponding author upon reasonable request.
